# Exploratory segmentation of selected cardiovascular hospital services in Chile using DRG-based normalized indicators

**DOI:** 10.3389/frhs.2026.1859518

**Published:** 2026-08-26

**Authors:** José Rodríguez, Manuel Vargas, Pilar Cos, Andrés Viveros

**Affiliations:** 1 Facultad de Ciencias Económicas y Administrativas, Universidad Católica de la Santísima Concepción, Concepción, Chile; 2 Escola de Doctorat, Universitat de Lleida, Lleida, Spain; 3 Departamento de Ingeniería Industrial, Universidad de Santiago de Chile, Santiago, Chile

**Keywords:** Chilean public health system, cluster analysis, diagnosis-related groups (DRG), hospital performance, principal component analysis

## Abstract

**Objective:**

To assess the use of DRG-based normalized indicators in supporting an exploratory analysis of inter-hospital variation in specific cardiovascular services within the Chilean public system from 2019 to 2023, employing routinely collected administrative data and confining interpretation to descriptive comparisons within the examined sample. The study was designed as descriptive and hypothesis-generating, not to validate hospital performance measures.

**Methods:**

An analysis was conducted of 141,893 adult discharges from the departments of Cardiology, Cardiovascular Surgery, and Peripheral Vascular Surgery in 54 hospitals within the Chilean public system. Relative throughput, severity, and mortality were summarized into three DRG-based normalized indicators as a function of coded complexity. The hospital-level means were standardized to z-scores prior to principal component analysis. Sampling adequacy for PCA was assessed using the Kaiser–Meyer–Olkin statistic (KMO = 0.6251) and Bartlett's test of sphericity (*p* = 1.00 × 10^−10^), which suggested performing exploratory analysis with caution. K-means clustering was then performed on the first two principal components with k = 4, n_init=50, and random_state=42 for reproducibility. Differences (specialty, sex, cluster) in the relative throughput proxy were assessed using the Kruskal–Wallis test and Dunn's *post hoc* tests.

**Results:**

The first two principal components accounted for 86.26% of the variability in the hospital-indicator matrix. K-means clustering resulted in four hospital clusters: cluster 0 (8 hospitals, 14.8%), cluster 1 (8 hospitals, 14.8%), cluster 2 (26 hospitals, 48.1%), and cluster 3 (12 hospitals, 22.2%). Distinct combinations of indicators emerged from the cluster means: cluster 1 had the highest ER (0.9633) and the lowest MR (0.0016), while cluster 3 had low ER (0.2465), high SR (2.3286), and the highest MR (0.0375). Kruskal–Wallis tests indicated significant differences in ER by specialty, sex, and cluster (all *p* < 0.001)

**Conclusions:**

The analysis suggests that DRG-based normalized indicators can be used to explore the segmentation of hospitals providing selected cardiovascular services within the Chilean public system. Their outputs should be interpreted as descriptive and hypothesis-generating rather than as validated measures of hospital efficiency or stand-alone evidence for resource-allocation decisions.

## 1 Introduction

In general, comparing hospital performance within national public health systems remains methodologically challenging, as hospitals vary in case complexity, service organization, and the constraints they face. These challenges are even more pertinent in an era of financial constraints and sustained demand, such that policymakers require hospital-level indicators that enable comparisons across hospitals while sufficiently accounting for clinical complexity at the individual hospital level. As such, this paper seeks to investigate whether dimensionless metrics based on DRGs can be used to describe differences among public hospitals in Chile that provide cardiovascular services from 2019 to 2023.

In Chile, the DRG is used to categorize hospital discharges for budgeting and financing. Although this framework standardizes case classification, resulting in higher-quality comparisons, it does not fully address the challenge of comparing hospitals that represent different types and levels of specialization, complexity, and size. Traditional measures such as crude mortality, mean cost, or simple productivity indicators might therefore be inadequate for depicting situations in which the aim is to contrast institutions with differing clinical workloads and service patterns. This limitation motivates the search for complementary indicators that allow more structured comparison across hospitals operating under heterogeneous conditions.

## 2 Background and literature review

The performance of  hospitals has been measured using different techniques, such as comparative approaches adjusted for case mix, productivity measures, and cost-based measures. Normalized ratios could help to serve  as a comparison tool for small and medium-sized hospitals in this larger body of literature. While  their interpretability is limited by the extent to which the selected variables are representative of clinically meaningful processes, their greatest potential value may lie in reducing direct reliance on the absolute scale.

DRG payment systems were initially created in the US to classify hospital output for billing, facilitate payment reform, contain expenditures, promote efficiency, and provide reimbursement mechanisms (1, 2). Initial studies emphasize their quality assurance potential beyond financial purposes, notably the auditing of outliers, exceptional cases where resource consumption lies above or below  expectations. The DRG-based payment methodology spread to both the public and private sectors of health care systems internationally, based on these early experiences. DRG-based systems then became dominant in Europe. DRG-based systems may support service quality and help contain hospital expenditure, keeping spending below GDP, compared with the US, according to comparative studies conducted in several European nations (3). Wide service coverage, doctors’ salaries, and readmissions are common features of European systems, which also use volume-control strategies to avoid incentives that increase unnecessary service volume. Additional comparisons demonstrate that institutional design remains crucial for aligning incentives with system goals and that clinical decisions affect revenues even when doctors receive salaries (4).

These conclusions are corroborated by empirical assessments from Germany. After G-DRGs were introduced in dermatology, one study found that length of stay significantly decreased and DRG valuations stabilized over time (5). The authors emphasized, however, that efficiency demands should not come at the expense of care quality. A more recent scoping review of DRG adoption in Germany and Switzerland found that, although there has been extensive policy discussion, only a small percentage of studies provide solid empirical evidence (6). A decrease in length of stay is one of the most significant findings, but the effects on other issues remain varied and warrant further investigation.

There are new difficulties when DRG is implemented in low- and middle-income nations. Evidence from low- and middle-income countries indicates that numerous nations implemented spending caps to reduce financial risk and adopted or modified imported DRG models, frequently following pilot programs (7). However, the execution was hindered by insufficient coding standardization, limited data access, and immature information systems. Increasingly, recent quantitative studies from China empirically support the deflationary effect of DRG reforms. One study showed, using propensity score matching and DiD models, that DRG reduced hospitalization costs for stroke patients by about 20% and reduced LOS and OOP  payments (8). Similarly, another study showed a significant reduction in inpatient costs across a wide range of complex surgical fields, associated with a more concentrated cost distribution, suggesting a  reduction in the number of extremely costly cases (9).

Specialty-specific analyses provide more evidence of the benefits  and drawbacks of DRG reforms. One study showed that DRGs shortened hospital stays for patients undergoing ophthalmic surgery, with stable clinical outcomes and reduced nursing costs (10). In contrast, another study showed that DRGs reduced costs  in TCM hospitals but also raised concerns about reimbursement and the preservation of TCM's unique clinical practices, increased hospitalization frequency, and alterations in treatment regimens resembling those of Western medicine (11). Evidence from Taiwan demonstrated that non-mainland Chinese hospitals were able to maintain the quality and financial viability of laparoscopic cholecystectomy under DRG systems, provided patient complexity was adequately captured in reimbursement groupings (12).

DRG outcomes are so dependent on the quality of the administrative data. Errors in ICD coding can skew DRG payments in favor of undercoding (lower pay) or overcoding (higher pay), as shown by evidence from China (13). For accurate hospital measurement and equitable repayment, that study identifies the need for automated, standardized data-quality protocols. An increasing number of people think that DRG systems became possible only due to technological innovation. One study analyzed potential contributions of artificial intelligence in industrial engineering automation, decision-making, and operational performance (14). These approaches are crucial to the healthcare industry, as they enable providers to allocate resources more effectively, estimate costs, and produce high-quality administrative data; however, they also raise issues regarding labor displacement and ethical governance. Public health administration literature has argued that reforms of hospital financing should be integrated into broader governance arrangements that support process management, quality enhancement, and outcome-based accountability (15). This bolsters the proposition that DRG systems *per se* are not sufficient instrument, but must be supported, by institutional reform, regulatory surveillance, and an excellent information infrastructure.

The literature indicates that DRG-type  payment systems are usually associated with decreased length of stay and mixed results for total hospital expenditure. Problems with data quality, distributional effects across patient types, unintended clinical incentives, and a  lack of robust longitudinal evidence remain widespread. These ambiguities  call for exploratory research that examines variation at the hospital level using routinely collected administrative data, while considering policy implications with caution.

While DRG-based systems have been extensively studied for financing and cost containment, their use for descriptive longitudinal segmentation of hospitals - particularly in middle-income countries like Chile - remains underexplored. Most existing studies focus on efficiency measurement using frontier methods (e.g., DEA) or on cost-containment outcomes, but few have examined whether simple ratio-based indicators derived from DRG data can generate interpretable and stable hospital profiles for descriptive monitoring. This study addresses this gap by applying a framework of dimensionless coefficients (ER, SR, MR) to describe inter-hospital variation in selected cardiovascular services and to generate exploratory hospital profiles through PCA and clustering, without claiming to validate efficiency measures. Specifically, we address three research questions: (1) Can DRG-based normalized indicators summarize inter-hospital variation in selected cardiovascular services? (2) Do these indicators support stable exploratory hospital profiles? (3) How should such profiles be interpreted, given their descriptive and non-validated nature?

Unlike DEA or SFA, the proposed indicators do not require specification of a production frontier, variable selection, or assumptions about returns to scale. Unlike simple raw metrics (e.g., crude mortality or mean length of stay), our ratio-based normalization reduces dependence on absolute hospital size and case-mix. Unlike complex composite indices, our indicators are transparent, easily computed from administrative data, and intentionally descriptive rather than evaluative. The framework complements existing approaches by offering a stable, longitudinal descriptive tool for hypothesis generation rather than replacing them.

## 3 Materials and methods

This study is the first to apply normalized ratios to examine whether ratios of DRG weight, length of stay, severity, and  mortality yield meaningful interpretations that indicate hospital variation. The purpose of the analysis is to remove the direct dependence on hospital volume and to enable meaningful comparisons across low-, medium-, and high-volume hospitals in cardiovascular subspecialties by expressing these measures as ratios. It is a pragmatic case for exploratory monitoring and not a statement of comprehensive performance measurement. Thus, rather than simply an application of engineering tools to health care, this viewpoint is best regarded as a crude initial analogy.

Using non-identified DRG records from public hospitals in Chile, the study is designed as an exploratory observational study with pooled discharges from 2019 to 2023. The three ratio-normalized outcome measures, ER, SR, and MR, were computed from routinely collected administrative variables and then combined at the hospital level for hospital-level analysis. These markers were never designed to validate adjusted clinical performance, but rather to serve as a structured digest of the variation observed among hospitals for select cardiovascular procedures. 2.1. Construction of the coefficients

The three coefficients were derived directly from standardized variables in the DRG records, leveraging their operational and comparative nature. The coefficients are defined as follows:

- Relative Efficiency (ER):

ER = (Diagnosis relative weight)/ (Length of stay (in days))

This coefficient was used as a descriptive proxy for throughput, relating DRG-based case weight to length of stay. Higher values indicate that, within the selected specialties, a hospital discharge combined greater coded complexity with fewer bed-days. However, this indicator may also be influenced by discharge practices, coding patterns, referral structure, and differences in case selection. For that reason, it should not be interpreted as a validated stand-alone measure of hospital efficiency.

b. Relative Severity (SR):

SR = (Clinical severity level)/ (Diagnosis relative weight)

This indicator reflects clinical complexity adjusted by DRG weight, allowing for comparison of case severity in proportion to the assigned weight. It is useful to differentiate hospitals treating more critical cases, even when the total volume is similar.

c. Relative Mortality (MR):

MR = (Number of discharges due to death)/(Diagnosis relative weight)

Relative Mortality (MR) was used here as a normalized, outcome-related indicator that expresses in-hospital mortality (deaths occurring during the hospital stay) relative to the DRG-based case weight. This definition corresponds to the TIPOALTA field in the FONASA database, which indicates death at hospital discharge. This is distinct from other mortality definitions, such as 30-day post-operative mortality (e.g., STS criteria). Its interpretation is necessarily cautious because the metric depends on how well the assigned DRG weight captures underlying clinical complexity. Accordingly, MR should be understood as a descriptive comparative indicator within the study sample rather than as a validated measure of risk-adjusted hospital quality. Table 1 presents detailed descriptive statistics for the Relative Efficiency (ER) indicator.

**Table 1:** presents detailed descriptive statistics for ER.

Statistical	Relative efficiency
Minimum	0.0
25th percentile (Q1)	0.114
Median (Q2)	0.248
75th percentile (Q3)	0.543
Maximum	11.704
Average	0.454
Standard deviation	0.573
Coefficient of variation (%)	126.1

These three normalized metrics were used to support structured comparison across hospitals while reducing direct reliance on the absolute scale. However, they do not eliminate the influence of contextual factors such as referral patterns, coding practices, service organization, or resource availability. Accordingly, they should be interpreted as exploratory comparative indicators derived from anonymized DRG records rather than as validated context-free measures of hospital performance.

The indices described above are summarized in the following Table 2.

**Table 2:** Summary of indicators used.

Index	Formula	Description	Observed range
ER	IR_29301_PESO/ESTANCIA	Relationship between complexity and hospital stay	0.0000 – 11.7045
SR	IR_29301_SEVERIDAD/IR_29301_PESO	Severity adjusted for complexity	0.0000 – 6.2893
MR	(1 if TIPOALTA = ‘FALLECIDO’, else 0)/PESO	Mortality weighted by complexity	0.0000 – 2.5320

The theoretical rationale for this approach draws on three strands of literature. First, the proposed ratios draw on two related methodological ideas: comparative efficiency analysis in health care, where ratios and frontier methods are used to relate outputs to inputs (16), and the engineering tradition of dimensionless coefficients, where normalization reduces dependence on absolute measurement units (17). Second, DRG weight has been validated as a robust proxy for expected resource intensity and clinical complexity (3, 4). Third, the VBHC framework (18, 19) emphasizes value as outcomes relative to costs, which aligns with our use of ER (complexity/length of stay) as a descriptive proxy. We emphasize that these indicators are not proposed as measures of technical or allocative efficiency, but rather as descriptive proxies for comparing temporal patterns across heterogeneous hospitals.

To support the interpretability of these indicators, we performed a convergent comparison using an independent input-oriented DEA model under variable returns to scale, applied to the same cardiovascular hospital subsample. The Spearman correlation between ER and DEA efficiency scores was 0.7991 (*p* < 0.001), and the within-hospital CV was 0.2418 (95% CI: 0.1642–0.2689). These results, summarized in Supplementary Table S3, support the use of ER as an exploratory proxy for efficiency patterns but do not constitute external validation of the criterion.

### 3.1 Sample, data collection, and cleansing

Anonymized National Health Fund of Chile (FONASA) DRG records (20) from 2019 to 2023 were used to create the study dataset. 4,709,438 discharges from all specialties and age groups were included in the original source. The analytical sample consisted of 141,893 records from 54 hospitals after the predetermined eligibility criteria—restriction to adult patients, selection of three cardiovascular specialties, minimum stay greater than zero, and complete information for the variables required to construct the indicators—were applied. Because the analysis was purposefully limited to a clinically specific subgroup rather than intended to represent all Chilean hospital discharges, the resulting retention rate was lower than in the entire DRG database. Table S1 shows the aforementioned information; it is available in the supplementary material.

Four of the 5,312 hospital discharges with a “DECEASED” status have a relative weight of zero, making it impossible to calculate the relative mortality rate (because the denominator was zero). Consequently, 5,308 deaths, or 99.92% of the valid cases, were used to calculate the relative mortality rate. Only these records fell within the observed range of 0 to 2.5013.

The public DRG system of the Chilean National Health Fund (FONASA) serves as the data source. The “Records after Filtering” were produced by applying the following filters in order:

1. Specialties: The study concentrated on the medical specialties of peripheral vascular surgery, cardiology, and cardiovascular surgery. The medical comparability of the procedures in each case served as the basis for selection.
2. Patients: Only adults were considered. The specific filter was ≥18 years of age, meaning this study focused on adult users of the public system.
3. Hospital stay: This is defined as the time elapsed between the patient's admission and discharge from the healthcare facility. The selection criterion was that all patients had to have stayed at least 1 day. To obtain this data, the filter considered hospital stays strictly greater than zero.
4. Missing Records: The database needed the columns IR_29301_PESO, IR_29301_SEVERIDAD, ESTANCIA, TIPOALTA, COD_HOSPITAL, and ESPECIALIDAD_MEDICA to calculate the dimensionless indices ER, SR, and MR. It was required that the fields for each health system user were correct and complete.
5. Retention Rate: This shows the ratio of the original records to the records after filtering (4,709,438, 141,893). This lessens the effects of missing data, as recent research suggests (21).

The data were processed to define a single entry per call for service per officer and to standardize officer names across all records containing them. This, in turn, made our analytic sample more uniform and less subject to avoidable error. Nevertheless, this data cleansing process is to be regarded as an en route stabilization of the descriptive statistics rather than an enhancement of inferential robustness.

After cleaning the data, hospital-level means of standardized ER, SR, and MR (z-scores) were used in multivariate analyses. For PCA, the Kaiser–Meyer–Olkin measure and Bartlett's test of sphericity were used. The overall KMO was 0.6251, an acceptable level for exploratory dimensionality reduction, and Bartlett's test was also significant (*p* = 1.00 × 10^−10), suggesting that the correlation matrix is not an identity matrix. To minimize sensitivity to local minima in clustering when using random initial centers, k-means is run with 50 random initializations (n_init = 50) under a fixed seed (random_state = 42), thereby enhancing computational reproducibility.

The PCA was used only for visualization and dimensionality reduction. In our subsample, PC1 and PC2 accounted for 86.26% of the total variance (PC1: 58.27%; PC2: 27.98%). The loading patterns revealed that ER and SR loaded in opposite directions on PC1 (ER: −0.6323, SR: 0.6488), and MR was the major contributor on PC2 (0.9028), suggesting that the three measures tap related but not fully overlapping constructs. As a sensitivity analysis, we also applied K-means clustering directly to the standardized indicators, without PCA. The cluster configuration was very similar to that of the PCA-based clustering (Adjusted Rand Index > 0.95), indicating that the segmentation pattern is robust with respect to the choice of dimensionality-reduction method (Table 1 in the Supplementary Material). Given the nature of an exploratory study, PCA was used primarily to aid visualization and enhance cluster interpretability, and its limitations are noted.

### 3.2 Methodological process

The process methodology followed these sequential steps.

#### 3.2.1 Data collection

The annual DRG files (2019–2023) were retrieved from the FONASA website. They had 4,709,438 discharge records. Each file had different technical characteristics (UTF-8 or UTF-16); the separator was inconsistent, and the internal organization was not standardized. It was processed in Python 3.10.11 (Visual Studio Code). The main libraries used were pandas, NumPy, Scikit-learn, matplotlib, seaborn, SciPy, and scikit-*post hoc*.

#### 3.2.2 Data cleansing and standardization

1. A data cleaning protocol was designed for each annual file, addressing aspects such as:
2. Correction of encodings (uniform conversion to UTF-8)
3. Homogenization of field separators (pipe |)
4. Conversion of misinterpreted numeric variables to text
5. Removal of incomplete or technically erroneous records
6. Validation of dates, ages, and key clinical variables (DRG weight, severity, mortality, hospital stay)
7. Filter by specialty: only Cardiology, Cardiovascular Surgery, and Peripheral Vascular Surgery.
8. Age filter: users aged 18 or older.
9. Hospital stay filter: longer than 0 days.

Complete Data Filter: no null values in the fields COC_HOSPITAL, ESPECIALIDAD_MEDICA, IR_29301_PESO, IR_29301_SEVERIDAD, ESTANCIA, TIPOALTA, and SEXO.

The applied filters ensured that the analyses were performed on a clinically homogeneous population with high-quality information. Supplementary Table S1 summarizes the source data, filtering process, retention rate, and final analytical sample used in the study.

**Figure 1:**
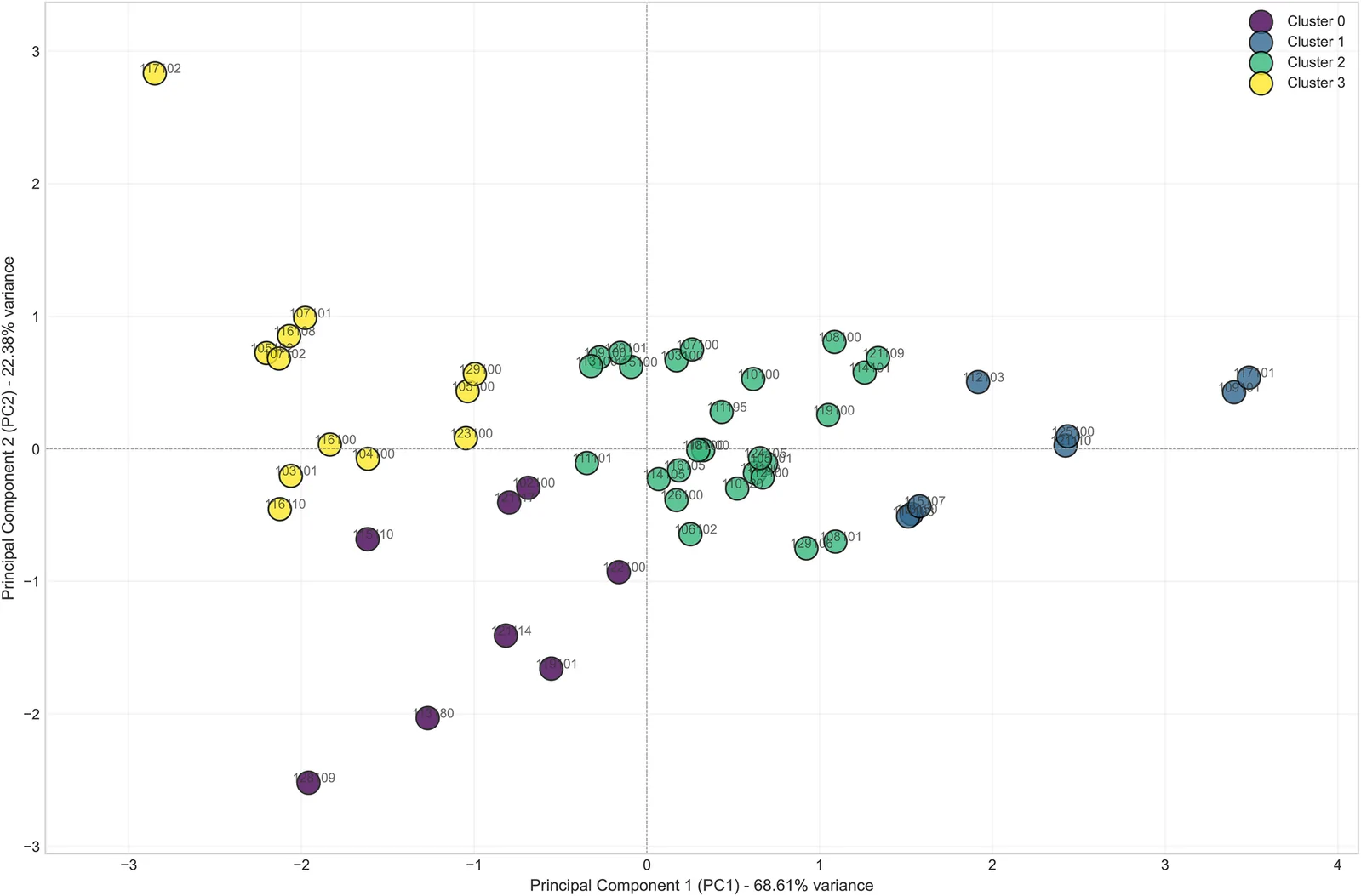
Principal component analysis (PCA).

**Figure 2:**
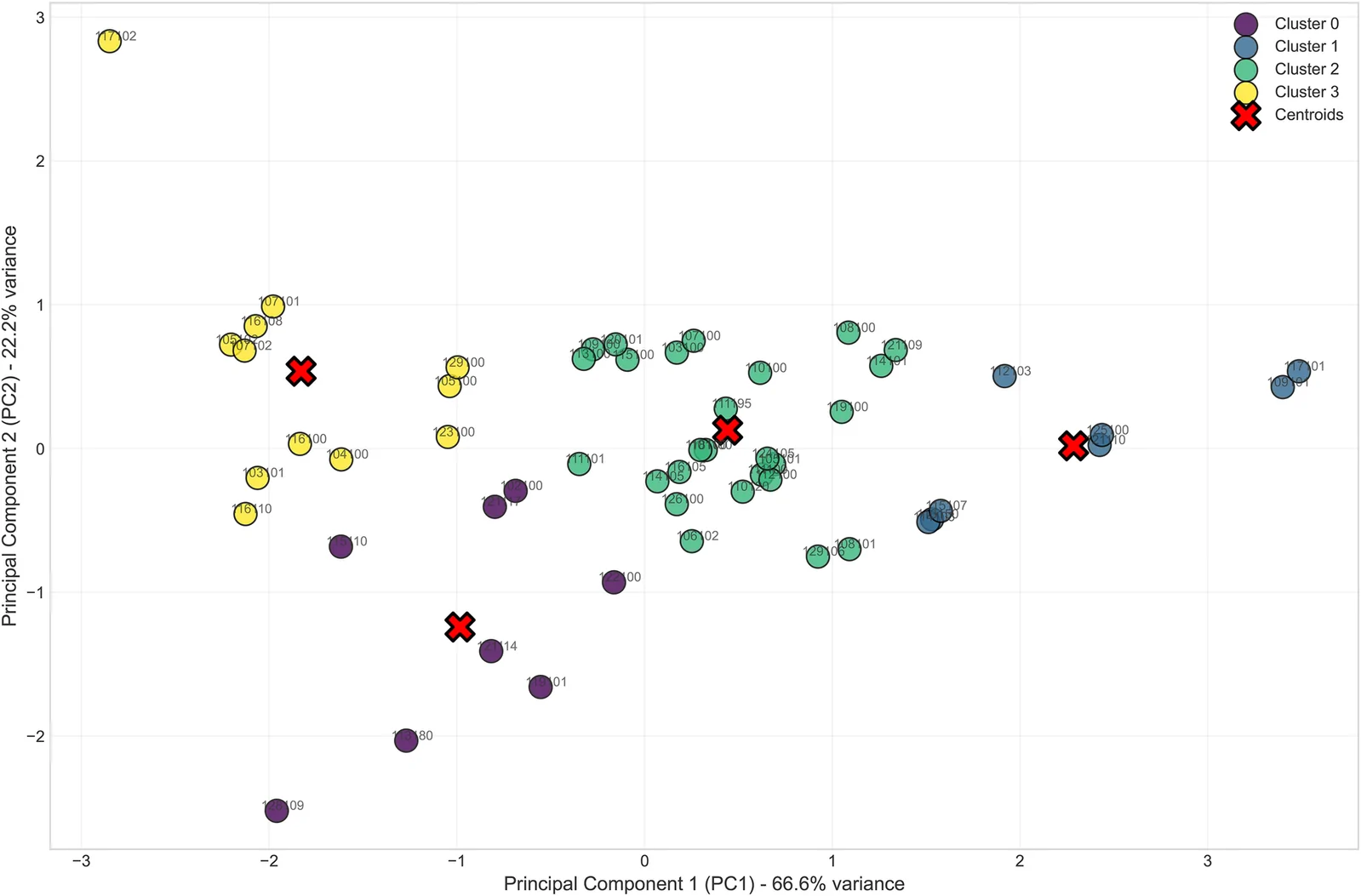
Distribution of hospitals on the PCA plane.

Steps performed in the processing and analysis:

1. Index calculations considered the hospital discharge rate.
2. Aggregation by hospital: calculations considered the average ER, SR, and MR for each of the 54 hospitals in the sample.
3. Data normalization (Z-score) was applied to the three variables.
4. Principal component analysis (PCA) was applied to the matrix formed by the 54 hospitals and the 3 variables shown in Figures 1 and 2.
5. K-means clustering was performed on the first two principal components with k = 4, as determined by the elbow and silhouette criteria. To improve stability, the algorithm was run with n_init = 50 and random state = 42, and the final partition corresponded to the solution minimizing within-cluster variance under those fixed reproducibility settings.
6. Generation of figures and tables.
7. Statistical tests:
  - Kruskal–Wallis test to compare ER between specialties, sexes, and clusters.
  - Dunn’s *post hoc* test with Holm correction was used for significant comparisons.

The selection of k = 4 was justified using three quantitative criteria. First, the elbow method showed an inflection point at k = 4. Second, the silhouette score for k = 4 was 0.412, and the Calinski-Harabasz index was 50.79, indicating moderate but acceptable cluster separation for exploratory analysis. Third, stability was assessed across 100 random initializations, yielding a mean Adjusted Rand Index of 0.9957 and a constant inertia of 36.42 (SD = 0.00), indicating that the four-cluster solution is highly reproducible. Additionally, time-series cross-validation confirmed that the clustering structure remained stable across temporal folds. These criteria support selecting k = 4 as a stable and interpretable partition, though external validation is pending. The full cluster-number sensitivity results are reported in Supplementary Table S3.

To evaluate clustering stability, the K-means procedure was repeated across 100 distinct random seeds. Agreement between solutions was quantified using the Adjusted Rand Index (ARI), and within-cluster inertia was monitored across runs.

Robustness and sensitivity analyses were conducted to assess the framework's exploratory reproducibility. These included a convergent comparison with DEA; clustering directly on standardized ER, SR, and MR without PCA; repeated K-means estimation across 100 random seeds; comparison of alternative numbers of clusters; time-series cross-validation using Random Forest; and sensitivity analysis across pre-pandemic, pandemic, and post-pandemic periods. These analyses are summarized in Supplementary Table S3.

## 4 Results

### 4.1 Descriptive statistics of the indices

At the record level, Relative Efficiency has a mean of 0.454 and a median of 0.248, with a range from 0.00 to 11.704. The coefficient of variation (CV) reaches 126.1%, indicating very high dispersion in operational performance across the hospitals. Relative Severity was observed to be more stable, with a mean of 1.521, a median of 1.169, a range of 0.000 to 6.289, and a coefficient of variation of 64.3%. Relative Mortality, by its nature, has a low mean of 0.020 and a median of 0.000; however, when only the cases with MR > 0 are observed, which are 5,308 records, the average rises to 0.532, and its CV is extremely high, reaching a value of 646.6%, reflecting the rarity of the event and the high variability in the complexity of the deceased patients.

### 4.2 Principal component analysis

Principal component analysis was applied to the hospital-level averages of ER, SR, and MR across the 54 hospitals in the sample. Adequacy testing supported exploratory use with caution: the overall KMO value was 0.6251, indicating moderate sampling adequacy, and Bartlett**’**s test of sphericity was significant (*p* = 1.00 × 10^−10^), indicating sufficient correlation among variables for PCA. The first two components explained 86.26% of the total variance (PC1: 58.27%; PC2: 27.98%), so a two-dimensional representation retained most of the variation in the three-indicator matrix. The loading structure suggests that PC1 was negatively associated with ER and positively associated with SR and MR, whereas PC2 was more strongly associated with MR. These components should be interpreted as statistical summary dimensions rather than latent constructs of hospital quality or efficiency.

### 4.3 K-means clustering: four hospital profiles

K-means on the first two PCs yielded four clusters of hospitals. The algorithm was run with n_init = 50 and random_state = 42 for reproducibility. The silhouette value (0.412) indicates modest cluster separation (not the strong isolation seen with Bootstrapping), and the Calinski–Harabasz index (50.79) is consistent with an internal  partition structure at an exploratory level. The cluster sizes were 8, 8, 26, and 12 hospitals, respectively.

**Figure 3:**
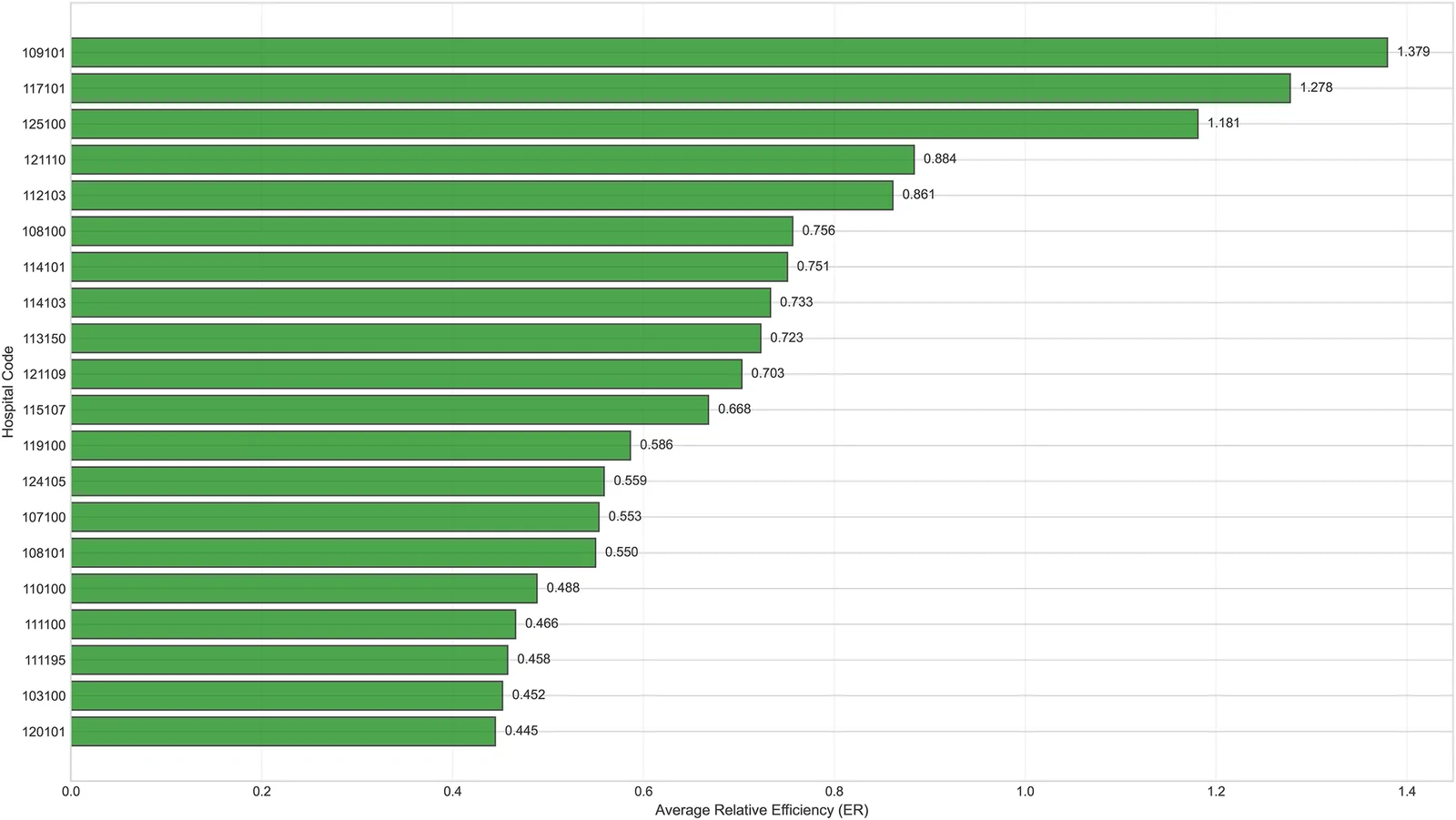
Ranking of the 20 hospitals with the highest average relative efficiency (2019−2023).

Figure 3 presents the top 20 hospitals with the highest average ERs over the study period. Since ER was considered a throughput indicator, using DRG weight divided by length of stay, this ranking should be read as a descriptive order among those included in the sample, not as a validated institutional efficiency hierarchy. In this subgroup, ER ranged from 0.78 to 1.38. Cluster analysis: Eight of these hospitals were in cluster 1, representing higher ER, lower SR, and lower MR, while most of the remaining hospitals were in cluster 2, denoting moderate indicator values. These trends imply diversity among high-ER hospitals, but they are not sufficient to pinpoint best practices or justify the direct policy transfer.

**Figure 4:**
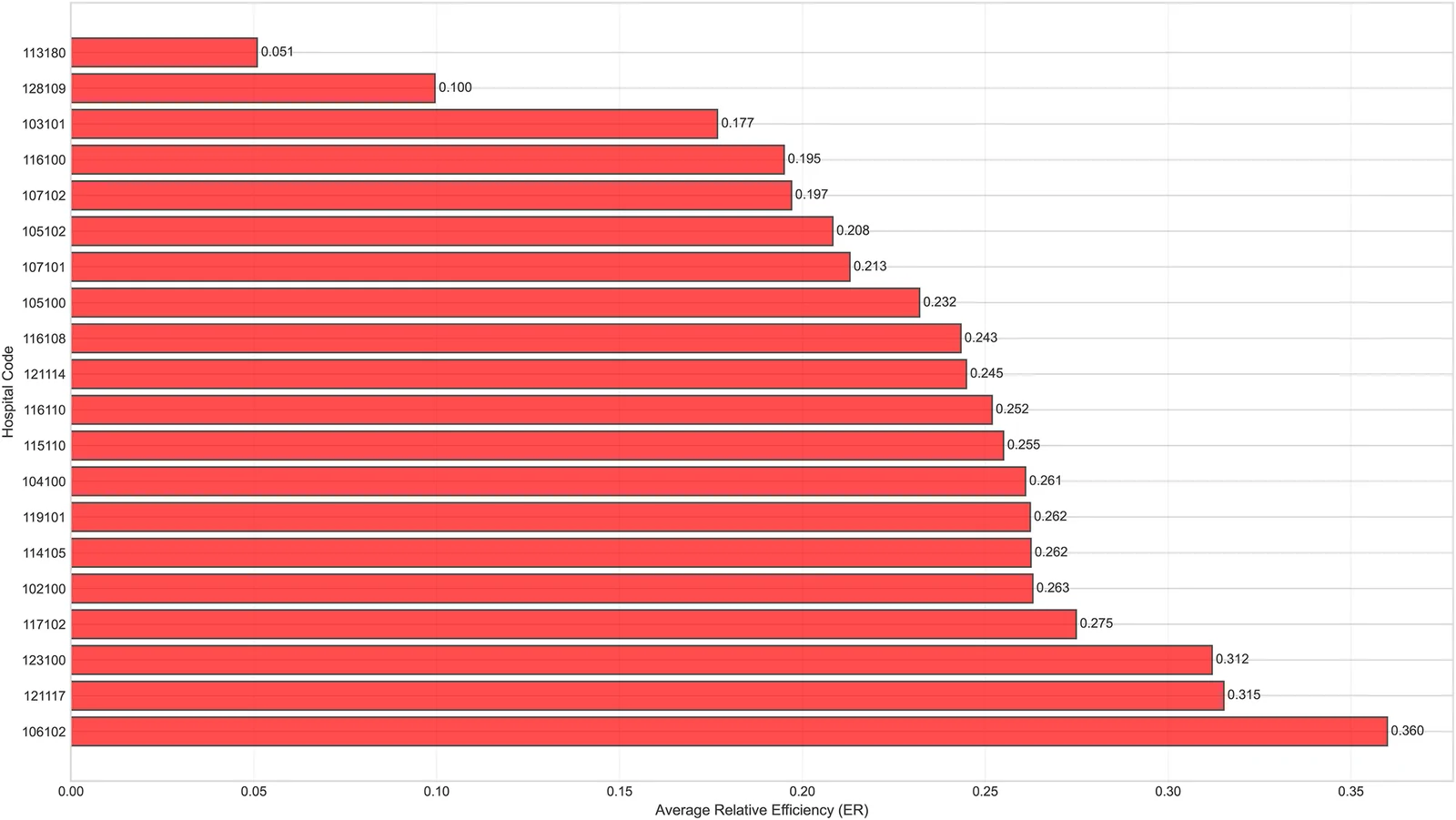
Ranking of the 20 hospitals with the lowest average relative efficiency (2019−2023).

Figure 4, Top 20 hospitals with the lowest average relative efficiency (2019–2023), illustrates the group of hospitals with the lowest relative throughput values. The twenty lowest-relative-efficiency hospitals had values between 0.16 and 0.27, which are substantially below the national median (0.25). The bottom-ranked hospital is still well short of the efficiency of 0.16, which is less than one-eighth of the top-ranked hospital's value.

These findings need to be interpreted with caution. The observed differences do not in themselves prove poor performance *per se* for these hospitals or isolate the influence of management-related factors. Instead, some hospitals appeared to have lower throughput proxies and higher case-severity profiles during the study period. This pattern may warrant further institutional evaluation; however, additional contextual factors are necessary before intervention priorities or policy suggestions are formulated.

The observed variation in the indicators indicates significant heterogeneity across hospitals in our sample. However, since the analysis was restricted to a limited number of cardiovascular subspecialties and the indicators were normalized by DRG, our results should be interpreted as a measure of within-service heterogeneity rather than as a ranking across the entire Chilean hospital network.

**Figure 5:**
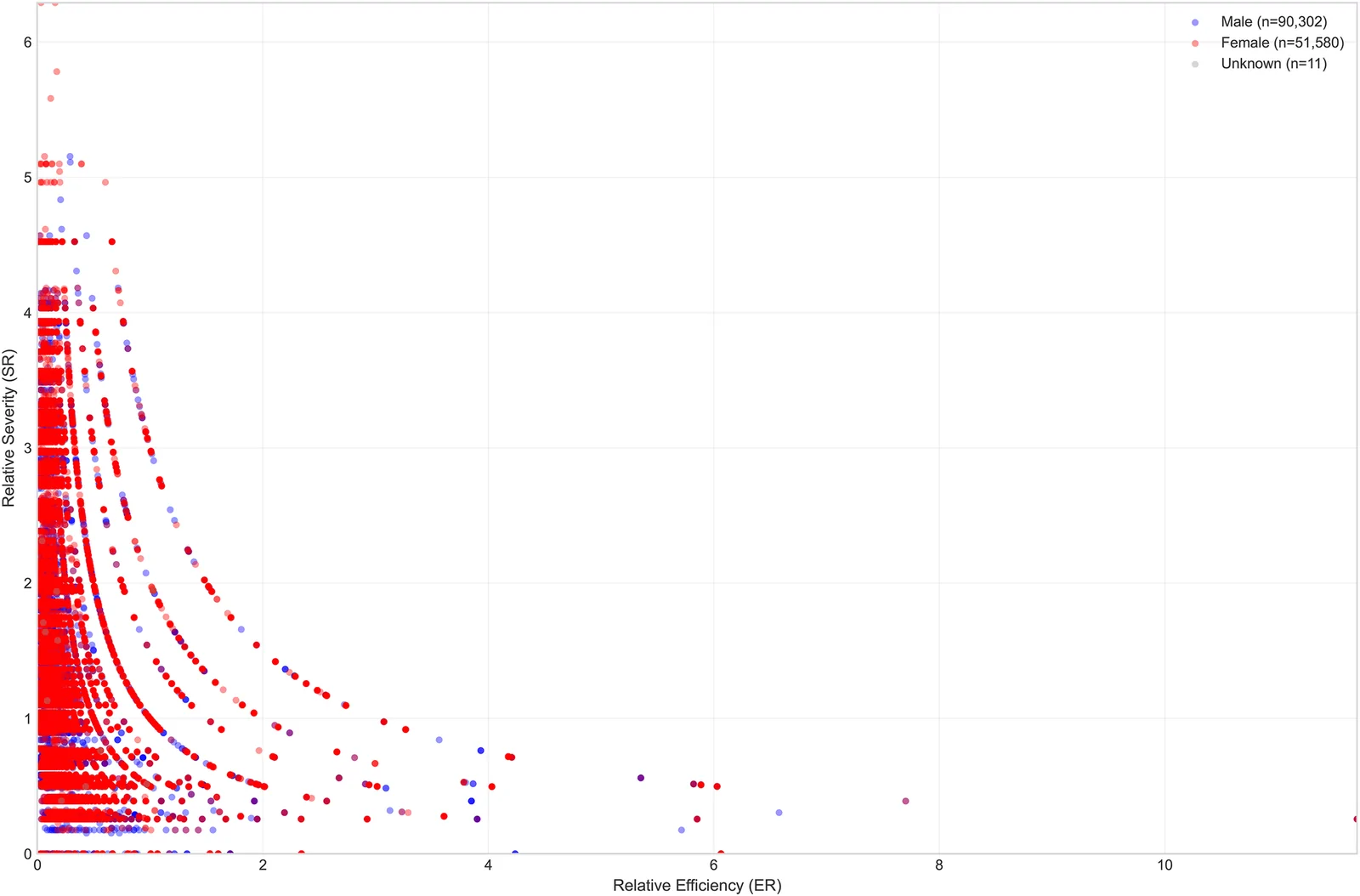
Distribution of hospital records in the PCA space according to patient sex.

A key benefit of the proposed method is a well-structured comparative framework that makes it easier to detect such differences. Figure 5 shows the projection of the 141,893 discharges on the first two principal components differentiating men (*n* = 90,302), women (*n* = 51,580), and 11 observations with unknown sex. There was no obvious visual separation between men and women, as the two groups largely overlapped within the component space and were situated in similar areas. At the macro. level, this is consistent with a pattern of similar efficiency, severity, and relative mortality profiles.

Although the Kruskal–Wallis test identified a statistically significant difference in the relative throughput proxy by sex, the magnitude of this difference should be interpreted with caution. With samples of this size, statistical significance may arise from modest distributional contrasts. Accordingly, the present analysis does not permit causal or normative conclusions regarding gender bias, differential treatment, or inequities in resource allocation.

Figure 5 supports two more limited interpretations. First, the overlap between male and female records in the component space suggests no strong visual separation in the joint distribution of the indicators analyzed. Second, the statistically significant difference in ER by sex should be treated as a signal of distributional difference within the sample, not as direct evidence of inequity, bias, or segregation.

1. No strong visual evidence of sex-based separation was observed in the indicators analyzed.
2. The statistical significance found should not be interpreted as evidence of segregation, but rather as a signal warranting specific studies using approaches designed to measure equity.

Table 3 summarizes the assignment of the 54 hospitals to four clusters derived from K-means on the first two principal components. The groups differed not only in size but also in their indicator averages: cluster 0 combined low ER (0.2360) with high SR (2.4178) and low MR (0.0085); cluster 1 showed the highest ER (0.9633), lowest SR (1.0720), and lowest MR (0.0016); cluster 2 presented intermediate ER (0.4709) and SR (1.3332) with low-to-moderate MR (0.0151); and cluster 3 combined low ER (0.2465), high SR (2.3286), and the highest MR (0.0375). These profiles are useful as exploratory summaries of variation within the selected specialties, but they should not be treated as stand-alone guidance for intervention.

**Table 3:** Individual assignment of each hospital to the corresponding cluster.

Cluster	Codes (hospital)	n	%^a^	Feature
0	102100, 113180, 115110, 119101, 121114, 121117, 122100, 128109	8	14.8	Low relative efficiency and high severity, low relative mortality.
1	109101, 112103, 113150, 114103, 115107, 117101, 121110, 125100	8	14.8	High efficiency, low severity, and minimal mortality
2	101100, 103100, 105101, 106102, 107100, 108100, 108101, 109100, 110100, 110120, 111100, 111101, 111195, 112100, 113100, 114101, 114105, 115100, 116105, 118100, 119100, 120101, 121109, 124105, 126100, 129106	26	48.2	Efficiency and moderate severity, relatively low mortality.
3	103101, 104100, 105100, 105102, 107101, 107102, 116100, 116108, 116110, 117102, 123100, 129100	12	22.2	Low efficiency, high severity, and higher relative mortality.

a Percentages are rounded; the exact values are 14.81%, 14.81%, 48.15%, and 22.22%, respectively.

### 4.4 Differences in relative efficiency according to specialty, sex, and cluster

The Kruskal–Wallis test showed significant differences in relative efficiency (ER) for the three variables analyzed:

To complement the *p*-values, we calculated effect sizes (eta-squared) using the formula eta-squared = H/(n – 1). The effect sizes were medical specialty: 0.0359 (small effect); sex: 0.00024 (very small effect); cluster: 0.0893 (moderate effect). These effect sizes indicate that, although all comparisons are statistically significant given the large sample size, the practical magnitude of the differences is small to moderate. The cluster effect is the most meaningful, supporting the segmentation as a descriptive tool. Therefore, statistical significance should not be equated with clinical or managerial relevance.

- Medical specialty: *χ*^2^ (2) = 5098.62; *p* < 0.001
- Sex: *χ*^2^ (1) = 33.92; *p* < 0.001
- Cluster: *χ*^2^ (3) = 12674.86; *p* < 0.001

Since the overall test was significant, Dunn's *post hoc* test with Holm's correction was applied for specialty and cluster (sex has only two categories, so the difference is direct).

Medical specialty: all three pairwise comparisons were significant (*p* < 0.001), indicating that the distribution of the relative throughput proxy differed across the three selected specialties. These differences support separate descriptive consideration of the specialties in the present dataset, although they do not, by themselves, establish the need for specialty-specific policy interventions.

**Figure 6:**
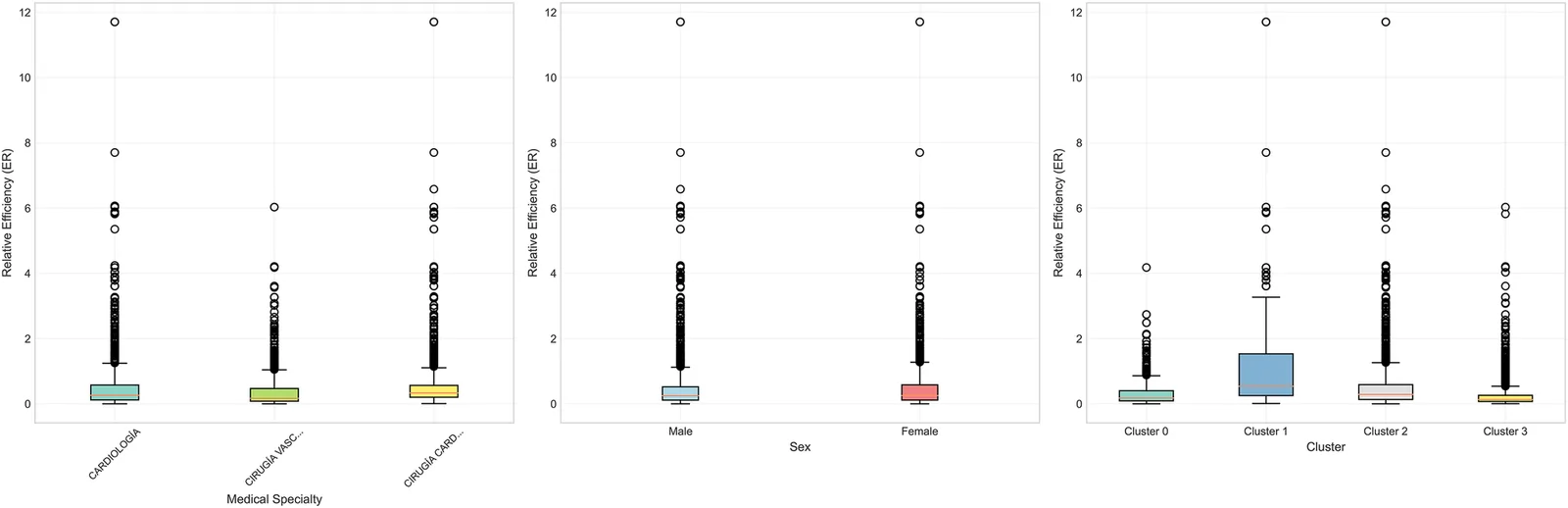
Distribution of relative efficiency (ER) by medical specialty, sex, and cluster. Boxplots compare the distribution of ER across the three grouping variables analyzed in the Kruskal–Wallis tests.

Cluster: all six pairwise comparisons were significant (*p* < 0.001), indicating that the ER distributions differed across the four cluster assignments. This finding supports empirical differentiation among the identified groups, but it should be interpreted as evidence of internal consistency in the segmentation rather than as external validation of the clustering framework. Figure 6 presents boxplots illustrating these differences.

### 4.5 System heterogeneity and coefficient of variation

The proxy relative throughput also had a high CV (126.1%), suggesting a wide spread of this measure across observations. This is in line with substantial heterogeneity in the chosen sample. However, the result should be interpreted as variation in the descriptive constructed measure rather than as direct evidence that the DRG payment system itself is creating unfairness.

### 4.6 Stability of the K-means solution

Robustness and sensitivity analyses (reported in Supplementary Table S3) supported the exploratory reproducibility of the segmentation. The four-cluster solution was highly reproducible across 100 random initializations from different starting centroids, suggesting stable convergence of the algorithm. The average Adjusted Rand Index with respect to the reference solution was 0.9957 (minimal value of 0.8938), and the average inertia did not vary between runs (36.42; SD = 0.00), indicating that the algorithm converges to the same partition regardless of the seed selection. To further evaluate stability, we conducted a triplet of analyses. First, as a consistency check, time-series cross-validation with Random Forest revealed that the clustering pattern was consistently replicated across each temporal fold (RMSE = 0.0617; Time Scale = 33.7%; ER_LAG1 = 28.5% var. imp.). Second, sensitivity analysis of clustering with and without PCA (ARI > 0.95). Third, results based on silhouette scores for k = 2 to 7 indicated that four (k = 4) is the optimal number of clusters, providing the best compromise between solution complexity and cluster separation (silhouette = 0.412). In sum, these findings suggest the exploratory reproducibility of the segmentation; however, reproducibility does not guarantee validity, and the results should not be taken as an external validation of a latent population structure.

## 5 Discussion

### 5.1 Comparison with international DRG-based performance studies

The cluster profiles should be interpreted as descriptive and hypothesis-generating, not as evidence of differential performance or quality. This is the first study to demonstrate that DRG-based normalized measures can be used to characterize variability among hospitals that perform selected cardiovascular procedures within the Chilean public system. The results should be considered a profiling exercise out of curiosity rather than a direct appraisal of hospital quality, efficiency, or management. This distinction is relevant, as international experience with DRG systems has indicated that DRG-based payment and classification systems may increase transparency and support cost containment (including by reducing LOS), but these results are biased by coding quality, case-mix adjustment, institutional context, and the specific design of the payment system.

The results are in line with international research that shows DRG systems can identify significant heterogeneity in hospital activity and resource consumption. European findings have highlighted the contribution of DRGs to enhancing hospital transparency and comparability, while research from low- and middle-income countries has identified implementation barriers related to data quality, coding standardization, and institutional capacity. New evidence from China also indicates that DRG-based reforms may reduce costs and length of stay in certain clinical fields, although the impact varies by specialty and may lead to perverse incentives. Within these lines, the present work contributes to the Chilean case by analyzing variability across hospitals in a few cardiovascular services using routinely collected administrative DRG data.

Nevertheless, the indicators suggested here are not to be considered in  the same way as official DRG payment results or audited performance measures. Relative Efficiency, Relative Severity, and Relative Mortality are descriptive ratios calculated from the administrative data. They reflect the patterns in throughput, coding severity, and in-hospital death relative to DRG weight, although they are not adjusted for hospital complexity, level of referral, infrastructure, human resources, or patient-level socioeconomic and clinical risk. Hence, the resulting clusters of hospital profiles should be considered for further exploration of institutions, rather than as a crystallized performance ranking of hospitals.

### 5.2 Methodological contributions and limitations of the proposed framework

The main methodological contribution of this study is the proposal of a simple and transparent framework for using DRG-based normalized indicators to describe inter-hospital variation. Unlike frontier-based efficiency methods such as DEA or SFA, the proposed indicators do not require specification of a production frontier, assumptions about returns to scale, or a formal input-output efficiency model. Instead, they provide a ratio-based descriptive approach that can be computed directly from routinely available administrative data. This makes the framework potentially useful for exploratory monitoring in settings where richer clinical, financial, or organizational datasets are not yet fully integrated.

As summarized in Supplementary Table S3, the convergent comparison with an independent input-oriented DEA model supports the interpretability of the proposed Relative Efficiency indicator as an exploratory proxy. The observed association between the proposed indicator and DEA efficiency scores suggests that both approaches may capture related patterns in the data. However, they remain conceptually different: DEA estimates relative efficiency under a formal frontier model, whereas the present framework summarizes DRG-based relationships between coded complexity, length of stay, severity, and in-hospital mortality. Therefore, the proposed indicators should be considered complementary to, rather than substitutes for, established efficiency and quality assessment methods. Importantly, this comparison does not constitute external criterion, construct, or predictive validation; the indicators should therefore be treated as administrative proxies for exploratory monitoring rather than as validated measures of hospital quality.

The PCA and clustering strategy also have an exploratory role. PCA was useful for summarizing the three normalized indicators and facilitating visualization, while K-means clustering identified four hospital profiles with distinct combinations of ER, SR, and MR. The stability analyses suggest that the clustering solution was not merely the result of a single random initialization. Nevertheless, reproducibility does not imply external validity. The moderate KMO value indicates that PCA was suitable for exploratory dimensional reduction, but not for strong latent-structure interpretation. Similarly, the four-cluster solution should be understood as an interpretable partition within this sample, not as evidence of a definitive underlying structure in the Chilean hospital system.

Several limitations should be emphasized. First, the analysis was restricted to Cardiology, Cardiovascular Surgery, and Peripheral Vascular Surgery, which improves clinical comparability but limits generalizability to other specialties. Second, the indicators were derived from administrative DRG records and therefore depend on the quality, completeness, and consistency of the coding. Third, observations were aggregated at the hospital level, which allowed us to compare institutions but at the expense of concealing potential within-hospital heterogeneity across services, clinical teams, or patient subgroups. While this aggregation was necessary for our descriptive objective of profiling hospitals, it may mask important variation that could inform more targeted management interventions. Future research should explore within-hospital heterogeneity using disaggregated data to better understand the sources of variation captured by the proposed indicators. Taken together, these limitations suggest that the study should be viewed as an exploratory evaluation of DRG-based normalized measures for selected services in the public system, rather than as a definitive assessment of hospital performance.

Additionally, the analysis is conducted at the hospital level and does not incorporate contextual covariates such as hospital complexity, geographic location (urban vs. rural), teaching status, bed capacity, specialist availability, or patient-level sociodemographic characteristics (e.g., socioeconomic status, rurality, environmental exposures). These factors may influence the proposed indicators independently of hospital management or operational performance. Therefore, the observed differences between hospitals and the resulting cluster profiles should be interpreted as descriptive patterns that generate hypotheses for further investigation, rather than as definitive assessments of institutional quality or efficiency. Future research should integrate these contextual variables to better isolate the drivers of variation in the proposed indicators.

### 5.3 Implications for healthcare management and policy

From a management perspective, the proposed framework may help identify patterns that warrant further investigation. Hospitals with similar profiles may share operational or clinical characteristics that are not immediately visible when using isolated indicators. For example, a profile combining low relative throughput, high relative severity, and high relative mortality may indicate a group of institutions requiring more detailed descriptive review. However, such profiles should not be interpreted as evidence of poor performance without additional contextual information.

The results also suggest that a single undifferentiated evaluative scheme may obscure meaningful heterogeneity among hospitals. Differences by specialty and cluster were more substantial than differences by sex, indicating that organizational and clinical context may be more relevant than demographic stratification for interpreting the proposed indicators. At the same time, statistically significant differences should not be equated with managerial or clinical relevance. In large administrative datasets, small distributional differences may become statistically significant, so effect sizes and contextual interpretation are essential.

The framework is also aligned with the broader logic of value-based healthcare, in the sense that it relates outcomes or activity measures to resource- or complexity-related denominators. However, the indicators used in this study are not full VBHC measures. They do not include patient-reported outcomes, long-term survival, readmissions, costs, or quality-adjusted outcomes. Therefore, their role is preliminary: they may support descriptive monitoring and hypothesis generation, but they should not be used as stand-alone evidence for resource allocation, hospital ranking, or payment reform.

For policy purposes, the main implication is that DRG data can support more granular monitoring of hospital heterogeneity, but only if interpreted cautiously. Before these indicators are used for decision-making, they should be externally validated, tested in other specialties, compared with clinical outcomes, and adjusted for institutional and patient-level context. Their greatest immediate value is as a screening tool for identifying patterns that warrant deeper investigation.

### 5.4 Temporal stability and the impact of the pandemic

Additionally, the study period (2019–2023) encompassed the COVID-19 pandemic, which may have affected coding practices, case mix, length of stay, and hospital operations in ways not fully captured by the proposed indicators. To assess this, we performed a sensitivity analysis comparing ER across pre-pandemic (2019), pandemic (2020–2021), and post-pandemic (2022–2023) periods. ER decreased significantly during the pandemic (−12.68%, *p* < 0.001) and partially recovered post-pandemic (+5.88%, *p* < 0.001) but remained below pre-pandemic levels. These sensitivity results are summarized in Supplementary Table S3 and support the need to interpret the pooled 2019–2023 estimates with caution.

At the same time, the clustering stability analyses suggest that the segmentation structure was reasonably reproducible within the available data. This means that the identified profiles may capture persistent descriptive patterns, but it does not prove that the same structure would remain unchanged under alternative time windows, clinical populations, or model specifications. Future research should explicitly model temporal trajectories, compare pre- and post-pandemic hospital profiles, and assess whether hospitals move between clusters over time.

Overall, this study should be understood as an exploratory application of DRG-based normalized indicators for hospital segmentation. The proposed framework provides a transparent way to summarize variation in selected cardiovascular services, but its outputs require cautious interpretation. Further work should validate the indicators externally, incorporate contextual covariates, examine within-hospital heterogeneity, and evaluate temporal stability beyond the pandemic period.

## 6 Conclusions

This exploratory study shows that selected cardiovascular services in Chilean public hospitals displayed heterogeneous clinical and utilization profiles during 2019–2023. The proposed DRG-based normalized indicators provide a transparent way to summarize patterns in throughput, severity, and in-hospital mortality using routinely collected administrative data. However, ER, SR, and MR should be interpreted as descriptive and hypothesis-generating proxies rather than as externally validated measures of hospital efficiency, quality, or performance. The clustering results may help identify patterns that warrant further investigation, but they should not be used as stand-alone evidence for ranking hospitals, allocating resources, or making policy decisions without external validation and contextual information.

Great variability was observed throughout the system under study. In particular, the high coefficient of variation (126.1%) for ER indicates a large spread of values for the throughput proxy across institutions. Four statistically distinct groups of hospitals emerged. One group exhibited relatively favorable values on all three measures, while another exhibited low relative throughput, high relative severity, and higher relative mortality. The remaining groups showed intermediate or mixed results.

These findings imply that a general, undifferentiated evaluative scheme could miss meaningful institutional heterogeneity within the service fields analyzed. The differences observed by specialty, cluster, and—though more modestly—sex suggest that future evaluations could be improved by considering clinical and organizational context, rather than by analyzing summary indicators pooled across these constructs.

Comparing hospitals using DRG-based information alone may obscure differences that become visible when multiple normalized indicators are examined together. Therefore, the current results support the cautious use of complementary indicators for planning, monitoring, and organizational evaluation, with the caveat that reforming the financing system would require further substantiation and policy discussion beyond the scope of this investigation.

The results should be taken as descriptive and hypothesis-generating rather than as providing direct proof of efficiency or causality-related variations in performance. However, the study shows how administrative data can be used to develop complementary tools that capture heterogeneity within institutions and guide decision-making.

Broadly, the protocol seems technically implementable in the administrative dataset, as it relies on readily available administrative data and employs conventional multivariate methods. The applicability of the tool to other contexts in Latin America is a matter for further research and would depend on local coding practices, data quality, and governance models.

## Data Availability

The original contributions presented in the study are included in the article/Supplementary Material, further inquiries can be directed to the corresponding author.
