# Supplementary material for: Exploratory segmentation of selected cardiovascular hospital services in Chile using DRG-based normalized indicators
Source: Front Health Serv. 2026 Aug 26;6:1859518. doi: 10.3389/frhs.2026.1859518 (PMC13553957; doi:10.3389/frhs.2026.1859518)
Supplement: Supplementary file 1 [file Table1.docx]

**Material Supplementary**

Table S1. Summary of extracted and processed data

| **Period** | **Source Files** | **Original records** | **Records after filters** | **Retention rate** |
| --- | --- | --- | --- | --- |
| 2019-2023 | Consolidated DRG Dataset | 4,709,438 | 141,893 | 3.01% |

Table S2. Kruskal-Wallis test of relative efficiency (ER) by specialty, sex, and cluster.

| **Analyzed variable** | **Groups (n)** | **Degrees of Freedom** | **χ²** | **p‑value** | **Dunn's post hoc (Holm's correction)** |
| --- | --- | --- | --- | --- | --- |
| Medical specialty | 3 | 2 | 5,098.62 | < 0.001 | 3 significant pairs (p < 0.001) |
| Sex | 2 | 1 | 33.92 | < 0.001 | Not applicable (only two groups) |
| Cluster | 4 | 3 | 12,674.86 | < 0.001 | 6 significant pairs (p < 0.001) |

Supplementary Table S3. Robustness and sensitivity analyses

| **Analysis** | **Purpose** | **Method** | **Main result** | **Interpretation** |
| --- | --- | --- | --- | --- |
| **DEA convergent comparison** | Assess whether ER is consistent with an established efficiency framework | Input-oriented DEA under variable returns to scale (VRS), applied to the same 54-hospital cardiovascular subsample. Spearman correlation with bootstrap (n=1000) | Spearman ρ = 0.8527 (95% CI: 0.8002–0.8917, p < 0.001); within-hospital CV = 0.2418 (95% CI: 0.1642–0.2689) | Supports ER as an exploratory proxy consistent with DEA logic but does not constitute external criterion validation. |
| **K-means without PCA** | Test dependence on PCA dimensionality reduction | K-means clustering on standardized ER, SR, MR (no PCA); k = 4; compared with PCA-based solution | Adjusted Rand Index (ARI) > 0.95 | Cluster assignments were robust to the choice of dimensionality-reduction approach. |
| **Random initializations** | Assess algorithmic reproducibility | K-means repeated across 100 random seeds; n_init = 50 per seed | Mean ARI = 0.9957; min ARI = 0.8938; inertia = 36.42 (SD = 0.00) | High reproducibility across random starts; solution is not an artifact of a single initialization. |
| **Selection of k** | Evaluate whether k = 4 is reasonable | Elbow method; silhouette; Calinski-Harabasz index for k = 2–7 | Silhouette (k=4) = 0.412; Calinski-Harabasz = 50.79; elbow inflection at k=4 | k = 4 offered a reasonable balance between interpretability and cluster separation for exploratory analysis. |
| **Time-series cross-validation** | Assess temporal reproducibility of the clustering structure | Random Forest with time-series split (3 folds), using hospital-year panel data with available lagged ER values (n = 183 observations; 49 hospitals; 2020–2023). | RMSE = 0.0617; most important variables: ESTANCIA (33.7%) and ER_LAG1 (28.5%); high stability across folds | Supports temporal reproducibility within the available data; clustering structure is stable across temporal folds. |
| **COVID-19 sensitivity** | Assess influence of the pandemic period on ER | Comparison of ER across pre-pandemic (2019), pandemic (2020–2021), and post-pandemic (2022–2023); Mann-Whitney U tests | ER 2019 = 0.4928; ER pandemic = 0.4304 (−12.68%, p < 0.001); ER post = 0.4556 (+5.88%, p < 0.001); remained below pre-pandemic levels (−7.54%, p < 0.001) | Pooled 2019–2023 estimates should be interpreted cautiously; pandemic had measurable impact with partial recovery. |
